# Reptin is required for the transcription of telomerase reverse transcriptase and over-expressed in gastric cancer

**DOI:** 10.1186/1476-4598-9-132

**Published:** 2010-05-30

**Authors:** Wenjuan Li, Jiping Zeng, Qiao Li, Li Zhao, Tiantian Liu, Magnus Björkholm, Jihui Jia, Dawei Xu

**Affiliations:** 1 Department of Microbiology/Key Laboratory for Experimental Teratology of Chinese Ministry of Education, School of Medicine and School of Life Sciences, Shandong University, Jinan, PR China; 2 Department of Medicine, Division of Hematology, and Center for Molecular Medicine (CMM), Karolinska University Hospital Solna and Karolinska Institutet, Stockholm, Sweden

## Abstract

**Background:**

Telomerase is activated in oncogenesis, which confers an immortal phenotype to cancer cells. The AAA + ATPase Reptin is required for telomerase biogenesis by maintaining telomerase RNA (hTER) stability and is aberrantly expressed in certain cancers. Given its role in chromatin remodeling and transcription regulation, we determined the effect of Reptin on the transcription of the *telomerase reverse transcriptase *(*hTERT*) gene, a key component of the telomerase complex and its expression in gastric cancer.

**Results:**

Knocking down Reptin or its partner Pontin using small interfering RNA in gastric and cervical cancer cells led to significant decreases in *hTERT *mRNA, but *hTERT *promoter activity was inhibited in only Reptin-depleted cells. Reptin interacted with the c-MYC oncoprotein and its stimulatory effect on the *hTERT*promoter was significantly dependent on functional E-boxes in the promoter. Moreover, Reptin bound to the *hTERT *proximal promoter and the loss of the Reptin occupancy led to dissociation of c-MYC from the *hTERT *promoter in Reptin-depleted cells. Reptin inhibition dramatically impaired clonogenic potential of gastric cancer cells by inducing cell growtharrest and over-expression of Reptin was observed in primary gastric cancer specimens.

**Conclusions:**

The *hTERT *gene is a direct target of Reptin, and *hTERT *transcription requires constitutive expression of Reptin and its cooperation with c-MYC. Thus, Reptin regulates telomerase at two different levels. This finding, together with the requirementof Reptin for the clonogenic potential of cancer cells and its over-expression in gastriccancer and other solid tumors, suggests that Reptin may be a putative therapeutic target.

## Background

Telomerase, an RNA-dependent DNA polymerase responsible for telomere lengthening, is silent in most differentiated human cells due to the tight repression of its catalytic unit, *telomerase reverse transcriptase *(*hTERT*) at the transcriptional level [1-3]. Because activation of telomerase is a critical step for cellular immortalizationand transformation [1-4], the mechanisms that control the precisely regulated switch between repression and activation of the *hTERT *gene and telomerase represent a central question in cancer research. As a multi-unit complex, telomerase activity has been shown to be controlled at various levels [1-3,5-7], however, it remains incompletely understood how telomerase and hTERT are de-repressed in oncogenesis.

Reptin and Pontin, two related AAA + ATPases, exert important biological actions including chromatin remodeling, transcription regulation and DNA damage repair [8,9]. They interact with major oncogenic factors such as β-catenin and c-MYC [8-12]. A few studies have shown that Reptin and Pontin are over-expressed in liver and colon cancers, respectively, and implicated in the pathogenesis of these cancers [13,14]. More recently, Reptin and Pontin have been identified as the subunits of the telomerase complex and they are required for telomerase assembly or biogenesis through maintaining telomerase RNA (hTER) stability [15]. Taken together, Reptin and Pontin are likely involved in carcinogenesis by promoting telomerase assembly and/or other mechanisms. Given an intimate interaction between Reptin/Pontin and the oncogene c-MYC, and essential role for c-MYC in the trans-activation of the *hTERT*gene [1-3,8,9,16-19], we hypothesize that Reptin and Pontin may regulate telomerase by affecting hTERT transcription. In the present study, we addressed this issue in gastric and cervical cancer cells. In addition, to determine the functional consequence and clinical relevance, we studied the effect of Reptin on the clonogenicpotential of gastric cancer cells and Reptin expression in primary gastric cancer specimens.

## Materials and methods

### Cell lines, culture conditions and small interfering RNA (siRNA) treatment

Human gastric cancer cell lines AGS, BGC-823 and HGC-27, and cervical cancer cell lines HeLa and SiHa used in the present study were cultured at 37°C/95%air/5%CO_2 _in RPMI 1640 medium (Life Technologies, Paisley, Scotland, UK) containing 10% fetal calf serum, 100 units/ml penicillin, and 2 mM L-glutamine. For siRNA treatment, cells were incubated in 6-well plates (1.0 × 10^5^/well) overnight and were then transfected with siRNA using Lipofectamine2000 (Invitrogen, Carlsbad, CA, USA) according to the manufacturer's protocol. Chemical modified Stealth™ siRNA targeting Reptin, Pontin and control siRNA (Invitrogen) were 5'- GAG AUC CAG AUU GAU CGA CCA GCA A -3', 5'-CCA UGC UGU AUA CUC CAC AGG AAA U-3' and 5'- CCU ACA UCC CGA UCG AUG AUG UUG A -3', respectively.

### RNA extraction, reverse transcription (RT) and real-time quantitative PCR (rqPCR)

Total cellular RNA in cell lines and gastric tissues was extracted using the ULTRASPEC kit (Biotecx Lab., Houston, TX, USA) or Trizol kit (Invitrogen) according to the manufacturer's protocols. cDNA was synthesized using random primers (N6) (Amersham, Buckinghamshire, UK) and M-MLV reverse transcriptase. The sequences of the PCR primers were: Reptin: 5'-CGAGGAAGAAGATGTGGAGA-3' (Forward) and 5'-CACTTCTGTACCCTTGCGTT -3' (Reverse); Pontin: 5'- GGCATGTGGCGTCATAGTAGA -3' (Forward) and 5'- CACGGAGTTAGCTCTGTGACT -3' (Reverse); hTERT: 5'-CGGAAGAGTGTCTGGAGCAA-3' (Forward) and 5'-GGATGAAGCGGAGTCTGGA-3' (Reverse); and hTER: 5'-TCTAACCCTAACTGAGAAGGGCGTAG-3' (Forward) and 5'-GTTTGCTCTAGAATGAACGGTGGAAG-3' (Reverse); c-MYC: 5'-TACCCTCTCAACGACAGCAGCTCGCCCAACTCCT-3' (Forward) and 5'-TCTTGACATTCTCCTCGGTGTCCGAGGACCT-3' (Reverse). β2-microglobulin (β2-M) expression was used as a control for RNA loading and RT efficiency and amplified. rqPCR was carried out in an ABI7700 sequence detector (Applied Biosystems, Foster City, CA) using a SYBR Green kit (Applied Biosystems). Levels of hTERT, hTER, Reptin and c-MYC mRNA were calculated based on the threshold cycle (CT) values and normalization of human β2-M expression.

### Assessment of telomerase activity

Telomerase activity was assayed with a commercial Telomerase PCR ELISA kit (Roche Diagnostics Scandinavia AB, Stockholm, Sweden) according to the manufacturer's instruction. Total cellular proteins were extracted using CHAPS lysis buffer. For each assay, 0.5 μg of protein was used, and 26 PCR cycles were performed after the telomerase-primer elongation reaction. The PCR products were detected with an ELISA color reaction. The measured telomerase activity was expressed as absorbance [optimal density (OD) in arbitrary units].

### Immunoprecipitation (IP) and immunoblotting

Total cellular proteins were extracted with RIPA lysis buffer. For IP, cellular proteins (200 μg) were precipitated with 5 μg of polyclonal antibodies against c-MYC (Santa Cruz Biotechnologies, Santa Cruz). Five μg of rabbit IgG served as an isotype control in IP experiments. The precipitated material, or 20 μg of protein in case of western blot for Reptin and c-MYC analyses, was subjected to sodium dodecyl sulfate-polyacrylamide gel electrophoresis and transferred to a nitrocellulose membrane. The membranes were probed with the specific monoclonal antibody against Reptin (BD, Franklin Lakes, USA) or polyclonal antibody c-MYC (Santa Cruz Biotechnologies) followed by anti-mouse or rabbit horseradish peroxidase-conjugated IgG and developed with the enhanced chemiluminescent method (ECL, Amersham). β-actin immunoblot was performed as a loading control.

### Colony formation assay

Cells were transfected with control, Reptin or Pontin siRNA for 72 hours, and were then seeded into six well-plates (300 cells/well) and incubated for 10 days, as described [20]. Plates were stained with Giemsa and the number of colonies with > 50 cells was counted.

### Assay for the hTERT promoter activity in cells with Reptin or Pontin depletion

The hTERT promoter reporter plasmid p181^wt ^harboring the core promoter sequence of the hTERT 5'-flanking region and its mutant variant (p181^MYC-^) lacking the functional c-MYC motifs (E-boxes) were described previously [17,21]. Cells cultured in 24-well plates were first transfected with Reptin or Pontin siRNA using Lipofectamine 2000 (Invitrogen). The next day, transfection of p181^wt ^and p181^MYC- ^plasmids was performed. A Renilla reniformis luciferase-containing plasmid, which is under control of the thymidine kinase promoter, was co-transfected to monitor the transfection efficiency. Luciferase activity in the cell lysates was determined by using a dual luciferase reporter assay system (Promega, WI) 48 hours post-transfection of the promoter reporter, and the *hTERT *promoter-driven firefly luciferase activity was normalized to the thymidine kinase renilla activity and expressed as percentage of that in control cells.

### Chromatin immunoprecipitation (ChIP)

ChIP assays were performed as described previously [22,23]. DNA was cross-linked by exposing the cells to medium containing 1% (vol/vol) formaldehyde for 10 minutes at 37°C and then sonicated to make soluble chromatin with DNA fragments from 200 to 1000 base pairs. Antibodies against Reptin (BD), c-MYC (Santa Cruz Biotechnologies) and acetylated histone H3 (Millipore, Temecula, CA) were used to precipitate DNA fragments bound by their corresponding elements. In negative control assays, rabbit IgG were added. The protein-DNA complex was collected with protein A/G-Sepharose beads (Millipore), eluted, and reverse cross-linked. After treatment with protease K (Sigma), DNA was extracted with phenol-chloroform and precipitated with ethanol. The recovered DNA was re-suspended in Tris-EDTA buffer and amplified by PCR with following primer pairs: *hTERT *proximal promoter: 5'-CCAGGCCGGGCTCCCAGTGGAT-3' (Forward), and 5'-GGCTTCCCACGTGCGCAGCAGGA-3' (Reverse). To further control for nonspecific antibody binding, the 3' region of GAPDH gene was PCR-amplified in parallel using the following primers: 5'-AAAGGGCCCTGACAACTCTT-3' (Forward) and 5'-GGTGGTCCAGGGGTCTTACT-3' (Reverse).

### Cell cycle analysis

Cells transfected with control and Reptin siRNA were harvested at 48 hrs, fixed with 70% ethanol at + 4°C overnight and stained with RNAse A (0.5 μg)-containing Propidium Iodide (PI, 50 μg/ml). Cell cycle distribution was determined using flow cytometry with ModFit.

### Patient samples and immunohistochemistry

Paraffin-embedded primary tumor specimens and their matched normal tissues were obtained from 20 gastric cancer patients who were admitted to Shandong University Qilu Hospital during 2007 [20,24]. The tissue specimens were de-paraffinized and rehydrated and the antigens retrieved in a microwave oven (0.01 M citrate buffer, pH 6.0). A mouse monoclonal antibody against Reptin (1:250) (BD) was then added onto the sections and incubated over-night at + 4°C. Goat anti-mouse IgG conjugated HRP and DAB staining (Vector Laboratories, Burlingame, CA, USA) were employed to visualize Reptin antibody binding. The slides were finally counterstained with hematoxylin. Mouse IgG instead of Reptin antibody was run in parallel as negative controls. A microscope (Olympus BX60, Tokyo, Japan) equipped with a digital camera (Sony DKC-5000, Tokyo, Japan) was employed for staining analysis. The result was expressed as percentage of staining-positive cells. The study was approved by the local Ethics Committee.

### Statistical analyses

The comparison of *hTERT *mRNA, promoter activity and telomerase activity, c-MYC expression and foci numbers between control and siRNA-treated cells was made using a Student's t-test or Mann-Whitney U-test. The difference in Reptin expression between tumors and adjacent normal gastric tissues as detected by immunohistochemistry was analyzed using Mann-Whitney U-test. A regression analysis was carried out to determine the correlation between hTERT and Reptin mRNA expression in gastric samples. All the tests were two-tailed and computed using SigmaStat3.1^® ^software (Systat Software, Inc., Richmond, CA). *P *values less than 0.05 were considered statistically significant.

## Results

### Reptin is required for constitutive hTERT mRNA expression in gastric and cervical cancer cells

**Figure 1:**
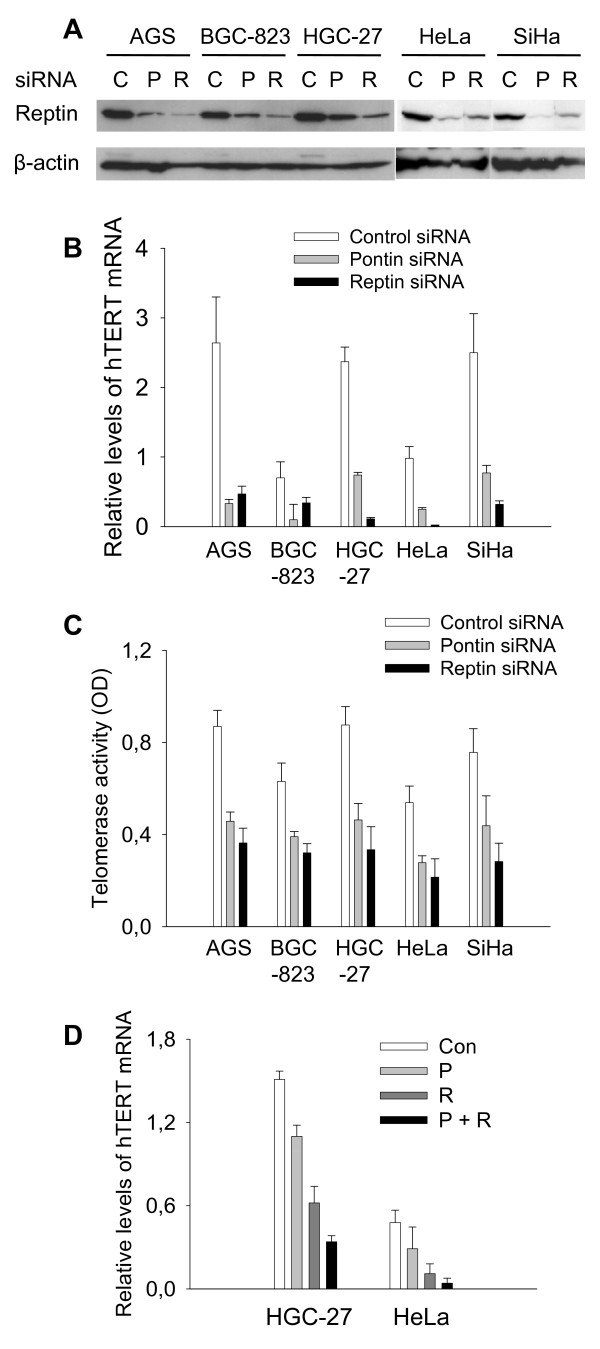
**Reptin depletion leads to reduced hTERT mRNA expression and telomerase activity in gastric and cervical cancer cells**. C, P and R: Control, Pontin and Reptin siRNA, respectively. Bars: Standard deviations. The results were obtained from three to four independent experiments. (A) Reptin protein expression in gastric and cervical cancer cells and efficiency of Reptin knocking down by siRNA. Cells were treated with control, Reptin or Pontin siRNA for 72 hours and then analyzed for Reptin expression by immunoblotting assay. (B) Reduced hTERT mRNA expression in Reptin or Pontin siRNA-treated cells. The same sets of cells from as above were used to determine hTERT mRNA using rqPCR. Relative levels of hTERT mRNA are shown. (C) Reduced telomerase activity in Reptin or Pontin siRNA-treated cells. Cellular proteins were extracted from the cells above and telomerase activity was assessed using a Telomerase ELISA kit. The enzymatic activity was expressed as absorbance [optimal density (OD) in arbitrary units]. (D) Additive inhibitory effect on hTERT mRNA expression in cells treated with combination of Reptin and Pontin siRNAs. HGC-27 and HeLa cells were treated with Reptin or Pontin siRNA alone or both of them at the half concentration used in (B) and hTERT mRNA abundance was analyzed with rqPCR. Relative levels of hTERT mRNA are shown.

Reptin were expressed at substantial levels in a panel of gastric and cervical cancer cell lines by immunoblotting assays (Fig. 1a). We silenced Reptin in those cells using its specific siRNAs. Efficient inhibition of Reptin was verified (Fig. 1a). rqPCR results showed a significant decline in *hTERT *mRNA expression in all three gastric cell lines treated with Reptin sequence-specific siRNA (Fig. 1B). To determine whether this is cell type-specific, the effect of Reptin depletion on *hTERT *mRNA expression was further evaluated in cervical cancer cell lines including HeLa and SiHa and we obtained identical decline in *hTERT *mRNA as seen in gastric cancer cells (Fig. 1B, control vs Reptin siRNA, *P *< 0.01). Thus, we conclude that Reptin is required for constitutive hTERT mRNA expression in gastric and cervical cancer cells. Consistent with the observations by Venteicher et al [15], we found that telomerase activity was significantly reduced in these cells (Fig. 1C, *P *< 0.01 in all Reptin siRNA-treated cells vs their corresponding control siRNA-treated cells). Of note, the decline in telomerase activity was less impressive compared to that of hTERT mRNA in Reptin-depelted cells, likely due to a relative longer half-life of telomerase activity [6]. In addition, the level of hTER, the telomerase RNA template, was also decreased in cells treated with Reptin siRNA (data not shown).

Because it has been shown that Reptin and its partner Pontin mutually depend on each other for their stability and either Reptin or Pontin sequence-specific siRNA co-depletes both of them [15], we tested if this was the case in the present setting. Pontin siRNA treatment indeed led to diminished levels of Reptin protein expression (Fig. 1A), despite a constant level of Reptin mRNA (data not shown). Significantly reduced *hTERT *mRNA and telomerase activity was similarly observed in Pontin-depleted cells (Fig. 1B and 1C, control vs Pontin siRNA: hTERT mRNA, *P *< 0.01; telomerase activity, *P *< 0.05).

### Combined treatment of cells with both Reptin and Pontin siRNAs leads to an additive decrease in hTERT mRNA

Despite the fact that either Reptin or Pontin sequence-specific siRNA leads to co-depletion of both proteins, we treated HGC-27 and HeLa cells with both Reptin and Pontin specific siRNAs to examine their combined action on *hTERT *mRNA expression. For this purpose, we utilized half the concentration of each siRNA for transfection, which led to weaker but significant inhibition of *hTERT *mRNA expression by each of them (Fig 1D, control vs Pontin or Reptin siRNA, *P *< 0.01). The combination of both siRNAs resulted in an additive decline in *hTERT *mRNA expression compared to that in cells treated with either siRNA alone (Fig. 1D, Pontin or Reptin siRNA vs both of them, *P *< 0.05).

### Reptin depletion results in a decrease in hTERT promoter activity significantly dependent on c-MYC

**Figure 2:**
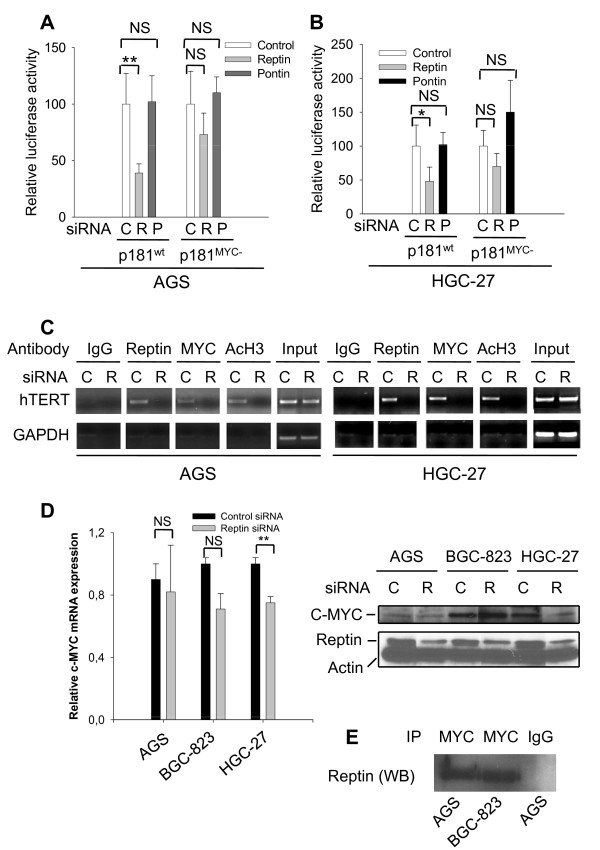
**Reptin cooperates with c-MYC to regulate the hTERT promoter activity**. (A and B) c-MYC-dependent inhibition of the hTERT promoter activity by Reptin depletion. AGS and HGC-27 cells were first treated with control (C), Reptin (R) or Pontin (P) siRNA and then transfected with either p181^wt ^or its c-MYC mutant variant p181^MYC-^. Luciferase activity was assessed using a dual luciferase detection kit 48 hours after transfection and relative luciferase activity was obtained from its normalization by co-transfected thymidine kinase renilla activity and expressed as percentage of that in control cells. Bars: Standard deviation. * and **: P < 0.05 and 0.01, respectively. Bars: Standard deviations. NS: Not significant. Three independent experiments were performed. (C) Concomitant presence or absence of Reptin and c-MYC on the hTERT promoter. AGS and HGC-27 cells were treated with control or Reptin siRNA and chromatin immunoprecipitation was then performed to examine the association between the hTERT promoter sequence and Reptin, c-MYC, or acetylated histone H3. (D) c-MYC expression in Reptin-depleted gastric cancer cells as determined by using rqPCR (Left panel) and western blot (Right panel). NS: Not significant and **: P < 0.01. (E) Physical interaction between Reptin and c-MYC as demonstrated using immunoprecipitation. Cellular proteins derived from AGS and BGC-823 cells were precipitated by a c-MYC antibody followed by detection using the antibody against Reptin. Rabbit IgG was used as a negative control. Two independent experiments were performed with similar results.

To probe whether Reptin and Pontin inhibited *hTERT *expression at the transcription level, we examined the effect of Reptin and Pontin depletion on the *hTERT *promoter activity. For this purpose, an hTERT reporter construct (p181^wt^) [17] that harbors the hTERT proximal promoter sequence were transfected into Reptin- and Pontin-specific siRNA-treated AGS and HGC-27 cells. Compared to that in control cells, Reptin depletion led to a significant reduction in the p181^wt ^-driven luciferase activity (Fig. 2a and 2B). In contrast, p181^wt ^activity was slightly increased in the cells treated with Pontin siRNA. Because Reptin has been shown to cooperate with c-MYC in regulating gene transcription [11,25,26], we further determined whether this effect of Reptin depended on c-MYC. We assessed the luciferase activity driven by the p181^wt ^variant p181^MYC- ^where two c-MYC binding motifs (E-boxes) on the promoter were inactivated by mutagenesis [17]. The inhibitory effect of Reptin depletion on the p181^MYC- ^activity became substantially weaker than on p181^wt ^(Fig. 2A and 2B), suggesting that Reptin regulates the hTERT promoter activity in a MYC-dependent fashion. Again, Pontin inhibition increased the p181^MYC-^activity even more compared to that of p181^wt^, especially in HGC-27 cells (Fig. 2A and 2B).

### Reptin occupies the hTERT promoter and its depletion abolishes the association of both Reptin and c-MYC with the hTERT promoter

The ChIP assay revealed that the *hTERT *promoter was bound by Reptin in AGS and HGC-27 cells, and such an association disappeared when Reptin was knocked down (Fig. 2C). We further found the concomitant absence of c-MYC on the promoter upon Reptin depletion ((Fig. 2C). Moreover, the acetylation of histone H3 in the hTERT promoter region was reduced in Reptin-depleted cells (Fig. 2C), suggesting a condensed chromatin structure consistent with lower transcription activity of the *hTERT*gene [27-29].

Undetectable MYC occupancy on the *hTERT *promoter may result from either down-regulation of c-MYC, or an impaired binding ability. To distinguish between these two scenarios, we determined c-MYC mRNA and protein levels in Reptin-depleted cells. Reptin siRNA-treatment led to a significant down-regulation of c-MYC expression in HGC-27 cells but not in AGS and BGC-823 cells (Fig. 2D). These results indicate defects in MYC binding to target promoters in the absence of Reptin.

### Reptin interacts with c-MYC

An interaction between Reptin and c-MYC has been observed in cells from different species [11,25,26], which is required for the transcriptional regulation of c-MYC target genes. To investigate whether this is the case in gastric cancer cells, we immunoprecipitated c-MYC-bound proteins using a c-MYC specific antibody and then performed immunoblotting analyses with an antibody against Reptin. The IP result demonstrated that Reptin was physically associated with c-MYC (Fig. 2E). Reptin and Pontin also interacted with each other in these cells (data not shown).

### Reptin-depleted gastric cancer cells show impairment of the clonogeneic potential and cell growth arrest

**Figure 3:**
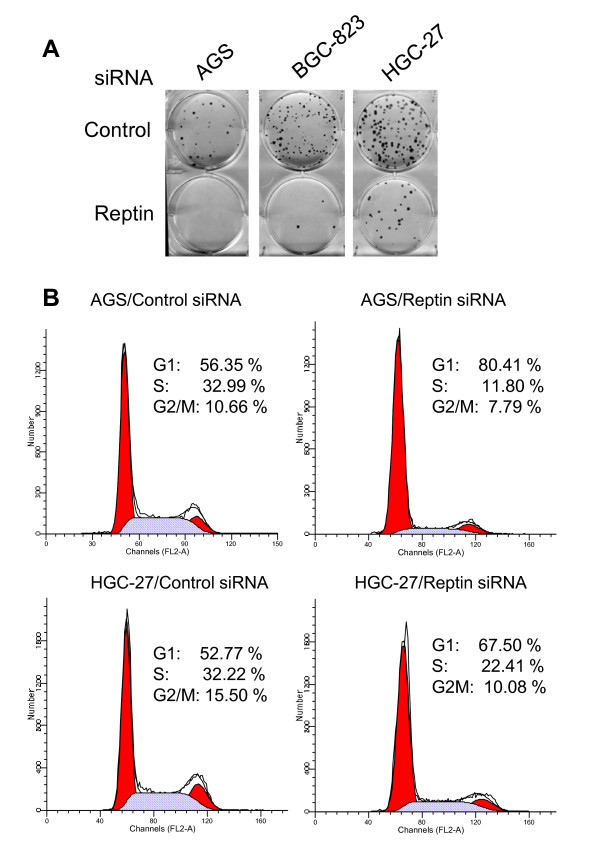
**Reptin depletion severely impairs the clonogenic potential and growth of gastric cancer cells**. (A) Cells were transfected with Reptin siRNA for 72 hours and then seeded into six-well plates (300 cells/well). After ten days, Giemsa staining was performed and the foci number was documented. Six independent experiments were performed. (B) AGS and HGC-27 cells treated with Control and Reptin siRNA were analyzed for cell cycle distribution using flow cytometry with ModFit. Two independent experiments were performed with similar results.

Telomerase or hTERT affects sustained cell proliferation through different mechanisms [30,31]. Given the findings described above, we sought to determine whether Reptin is required for the clonogenic potential of immortal cancer cells. To this end, we assessed the foci formation ability of the cells treated with Reptin siRNA. As shown in Fig. 3, Reptin depletion resulted in dramatic reduction in foci numbers of gastric cancer cells (Control siRNA vs Reptin siRNA in all three cell lines, *P *< 0.01. AGS (Mean ± SD): 42 ± 4 vs 0 ± 1; BGC-823: 152 ± 12 vs 4 ± 3 and HGC-27: 162 ± 41 vs 16 ± 11). Of note, AGS cells completely lost their clonogenic potential when Reptin was knocked down (Fig. 3A). Flow cytometry analyses demonstrated that these Reptin siRNA-treated cells underwent growth arrest at G1 phase (Fig. 3B). Lack of sub-G1 (Fig. 3B) and neglectable annexin V staining (data not shown) suggest that Reptin inhibition does not induce apoptosis of AGS and HGC-27 cells.

### Reptin is over-expressed in primary gastric cancer

**Figure 4:**
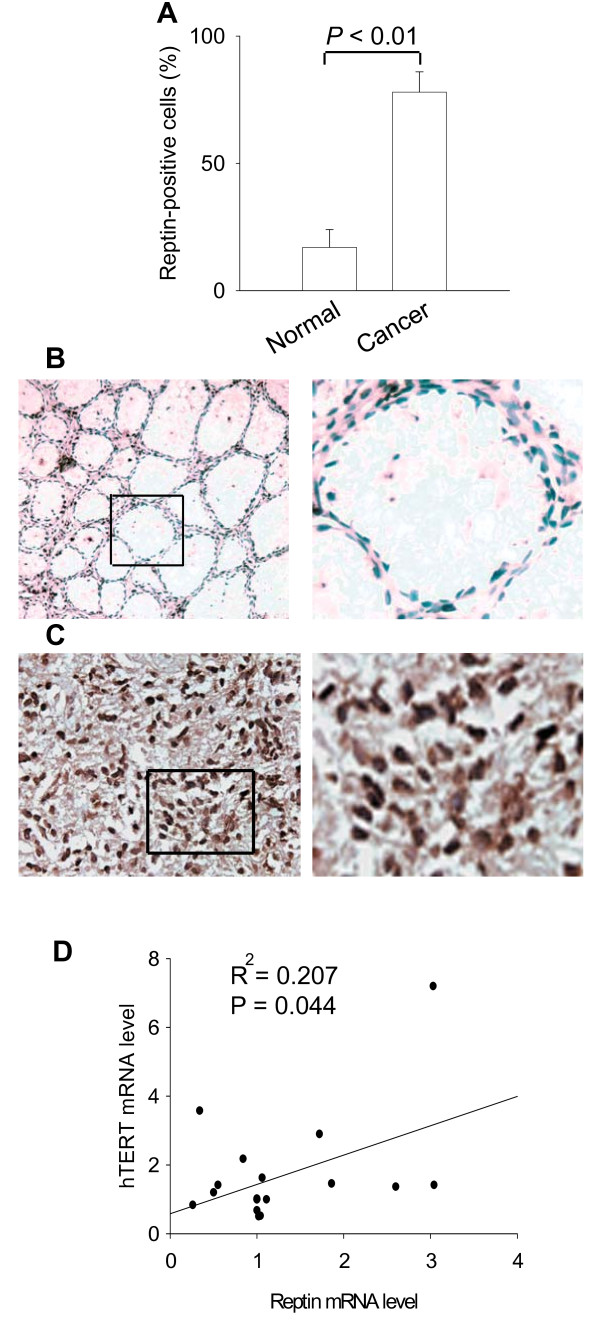
**Reptin is over-expressed in primary gastric cancer. Immunohistochemistry was performed to determine Reptin expression**. (A) Overall Reptin-positive cells in gastric cancer specimens and their matched normal tissues from 20 patients. (B) and (C) Representative of Reptin staining on normal and cancerous gastric tissues, respectively (Magnification × 400). Left panels: The enlarged areas of right panels in the rectangles. (D) Positive correlation between Reptin and hTERT mRNA levels in primary gastric tissues. The abundance of Reptin and hTERT mRNA in 20 normal and cancerous gastric tissues was assessed using rqPCR as described in Materials and methods.

We further assessed whether Reptin expression was altered in primary gastric cancer. Tumor specimens and their matched normal tissues derived from 20 patients with gastric cancer were analyzed by immunohistochemistry. In normal gastric mucosa, the majority of cells were negative for Reptin staining while a small fraction of cells had Reptin signals with variable intensity that were localized to the nucleus (Fig. 4A and 4B). However, in tumors, most cells were Reptin-positive with a much stronger staining (78 ± 8%, Mean ± SD) (Fig. 4A and 4C), which was significantly higher than those in matched normal tissues (17 ± 7%, Mean ± SD, tumors vs normal, *P *< 0.01). Tumor cells were in general stained homogenously, although there was heterogeneity to a certain extent. Similar to that seen in matched normal tissues, Reptin signals were predominantly distributed in the nucleus of tumor cells (Fig. 4C).

### Reptin mRNA expression is correlated with hTERT transcript level in gastric tissues

We finally determined whether Reptin and hTERT expression was associated with each other in primary gastric tissues. Twenty normal and cancerous gastric samples were analyzed for Reptin and hTERT mRNA abundance using rqPCR, and as shown in Fig. 4D, we found a positive correlation between them (R^2 ^= 0.202, P = 0.044), which indicates that Reptin over-expression likely has an *in vivo *stimulatory effect on the hTERT transcription.

## Discussion

Regulation of gene transcription is one important function of Reptin and Pontin, and likely achieved through two different mechanisms [8,9]. First, both Reptin and Pontin are integral subunits of different chromatin-modifying complexes such as ATP-dependent helicase Ino80- and Tip60-containing histone acetyltransferase (HAT) complexes. Second, they interact physically with various sequence-specific transcription factors, among which is the oncogene c-MYC [26,32]. Because MYC also recruits the Tip60 HAT complex to its target promoters [32,33], a link between Reptin and c-MYC apparently occurs at two different levels. With this background in mind, we determined the effect of Reptin and Pontin on hTERT expression. Here we show the *hTERT *gene is a direct transcription target of Reptin: (i) Knocking down Reptin led to a significant decline in *hTERT*mRNA expression and hTERT promoter activity; and (ii) Reptin occupied the *hTERT *proximal promoter. It was further observed that the functional c-MYC binding motifs on the hTERT promoter activity was required for Reptin depletion-mediated reduction of the promoter activity and that c-MYC failed to bind to the hTERT promoter without Reptin. Therefore, Reptin affects the hTERT transcription in a MYC-dependent manner. Our findings, together with the observation showing Reptin being a required factor for telomerase assembly, demonstrate that Reptin regulates telomerase activity through two different mechanisms. As telomerase has been suggested as a cancer therapeutic target, manipulating Reptin may be a novel approach for telomerase inhibition in cancer cells.

Reptin and Pontin are known to antagonize each other in regulating transcription of certain target genes [12,34-36]. It is not surprising to identify a differential effect on hTERT transcription between Pontin and Reptin. Like Reptin, Pontin interacts with c-MYC and its depletion leads to diminished *hTERT *mRNA expression. Reptin protein level was concomitantly reduced when Pontin was knocked down in all the examined cells. However, depleting Pontin slightly increased rather than inhibited hTERT promoter activity, in sharp contrast to that seen in cells treated with Reptin siRNA. It is currently difficult to understand that Reptin and Pontin depletion inhibits *hTERT *mRNA expression on the one hand while each of them has different effects on the hTERT promoter activity on the other. Nevertheless, the results collectively indicate that Pontin and Reptin regulate *hTERT *mRNA expression through different mechanisms.

Reptin depletion led to a significant reduction in foci numbers of gastric cancer cells, indicating that constitutive expression of Reptin is essential for their clonogenic potential. Such defective clonogenesis may be attributable to down-regulation of hTERT expression and telomerase activity, impaired c-MYC function and/or down-regulation, and growth arrest taking place in Reptin-depleted cancer cells. Indeed, hTERT or telomerase inhibition compromises sustained proliferation and tumorigenic ability of cancer cells [30,31] while the c-MYC-mediated cellular transformation is severely attenuated if without Pontin and Reptin [32]. Moreover, given diverse biological activities of Reptin [8], other unidentified mechanisms are likely involved in Reptin-mediated cancer cell growth and clonogenic ability.

Reptin and Pontin were previously reported to be expressed ubiquitously in normal human tissues [8], however, their enhanced expression has been observed in different types of cancer. We show here a general increase in Reptin expression in primary gastric cancer, potentially indicating its clinical relevance. Little is at present known about the regulatory mechanism of Reptin expression. The oncogene c-MYC, frequently dysregulated in gastric cancer [24], has been shown to significantly enhance Reptin expression [8]. We also observed the requirement of c-MYC for Reptin expression (WL and DX, unpublished data). Thus, the over-expression of Reptin observed in gastric cancer likely results from the elevated c-MYC level. In that setting, Reptin and c-MYC may form a positive feedback loop to regulate their expression and functional activities.

## Conclusion

In the present study, we demonstrated that Reptin was required for constitutive *hTERT *mRNA expression in gastric and cervical cancer cells. The depletion of Reptin led to significant decreases in *hTERT *mRNA and promoter activity by impairing c-MYC-dependent hTERT transcription. It was further shown that the Reptin-depleted cancer cells exhibited highly defective clonogenesis and cell cycle arrest. Finally, Reptin was observed to be over-expressed in primary gastric cancer. Collectively, Reptin may play an important role in the pathogenesis of gastric and other cancers, and targeting Reptin may thus be implicated in cancer therapy acting through inhibition of telomerase activity or other undefined mechanisms.

## Abbreviations

ChIP: Chromatin immunoprecipitation; hTERT: Human telomerase reverse transcriptase; IP: Immunoprecipitation; rqPCR:Real-time quantitative PCR; RT: Reverse transcription; siRNA: Small interfering RNA.

## Competing interests

The authors declare that they have no competing interests.

## Authors' contributions

WL, JJ and DX designed the study; WL, JZ, QL, LZ and TL performed experiments; WL, MB, JJ and DX analysed the data and wrote the paper. All authors read and approved the final manuscript.
